# AhpA is a peroxidase expressed during biofilm formation in *Bacillus subtilis*

**DOI:** 10.1002/mbo3.403

**Published:** 2016-09-28

**Authors:** Joelie V. Zwick, Sarah Noble, Yasser K. Ellaicy, Gabrielle Dierker Coe, Dylan J. Hakey, Alyssa N. King, Alex J. Sadauskas, Melinda J. Faulkner

**Affiliations:** ^1^ Department of BiologyBradley UniversityPeoriaILUSA; ^2^ Present address: School of Dental MedicineSouthern Illinois UniversityAltonILUSA; ^3^ Present address: Graduate School of Arts and SciencesGeorgetown UniversityWashingtonDCUSA; ^4^ Present address: School of MedicineSaint Louis UniversitySaint LouisMOUSA

**Keywords:** AbrB, AhpC, alkylhydroperoxide reductase, biofilm formation, oxidative stress, peroxide, peroxiredoxin, Spo0A, sporulation

## Abstract

Organisms growing aerobically generate reactive oxygen species such as hydrogen peroxide. These reactive oxygen molecules damage enzymes and DNA, potentially causing cell death. In response, *Bacillus subtilis* produces at least nine potential peroxide‐scavenging enzymes; two belong to the alkylhydroperoxide reductase (Ahp) class of peroxidases. Here, we explore the role of one of these Ahp homologs, AhpA. While previous studies demonstrated that AhpA can scavenge peroxides and thus defend cells against peroxides, they did not clarify when during growth the cell produces AhpA. The results presented here show that the expression of *ahpA* is regulated in a manner distinct from that of the other peroxide‐scavenging enzymes in *B. subtilis*. While the primary Ahp, AhpC, is expressed during exponential growth and stationary phase, these studies demonstrate that the expression of *ahpA* is dependent on the transition‐state regulator AbrB and the sporulation and biofilm formation transcription factor Spo0A. Furthermore, these results show that *ahpA* is specifically expressed during biofilm formation, and not during sporulation or stationary phase, suggesting that derepression of *ahpA* by AbrB requires a signal other than those present upon entry into stationary phase. Despite this expression pattern, *ahpA* mutant strains still form and maintain robust biofilms, even in the presence of peroxides. Thus, the role of AhpA with regard to protecting cells within biofilms from environmental stresses is still uncertain. These studies highlight the need to further study the Ahp homologs to better understand how they differ from one another and the unique roles they may play in oxidative stress resistance.

## 1 Introduction

Aerobically growing organisms must cope with the damaging effects of reactive oxygen species (ROS), including hydrogen peroxide (H_2_O_2_) and organic peroxides such as cumene hydroperoxide (CHP). To scavenge peroxides, cells produce numerous catalases and peroxidases. *Bacillus subtilis* potentially produces nine such peroxide‐scavenging enzymes: three catalases (KatA, KatB/KatE, and KatX), two alkylhydroperoxide reductases (AhpC and AhpA), two organic peroxidases (OhrA and OhrB), and two additional members of the AhpC/TSA family (thiol peroxidase Tpx and a putative bacterioferritin comigratory protein Bcp) (Antelmann, Engelmann, Schmid, & Hecker, 1996; Bagyan, Casillas‐Martinez, & Setlow, 1998; Bsat, Chen, & Helmann, 1996; Casillas‐Martinez & Setlow, 1997; Engelmann & Hecker, 1996; Fuangthong, Atichartpongkul, Mongkolsuk, & Helmann, 2001; Lu et al., 2008). Studies in *B. subtilis* demonstrate that its two primary peroxide scavengers are AhpC and the vegetative catalase KatA; strains lacking both *ahpC* and *katA* grow poorly under aerobic conditions (Broden et al., 2016; Bsat et al., 1996). Consistent with this, both *ahpC* and *katA* are expressed moderately well during exponential growth (Broden et al., 2016; Bsat et al., 1996; Faulkner, Ma, Fuangthong, & Helmann, 2012; Fuangthong, Herbig, Bsat, & Helmann, 2002). Furthermore, mutation of either *ahpC* or *katA* results in the increased expression of other proteins responsible for protecting cells against peroxides (Antelmann et al., 1996; Bsat et al., 1996; Chen, Keramati, & Helmann, 1995). Both *ahpC* and *katA* are regulated by the peroxide‐responsive repressor protein PerR (Helmann et al., 2001).

*Bacillus subtilis* AhpC belongs to the classical group of alkylhydroperoxide reductase (Ahp) proteins and possesses a dedicated reductase AhpF that is encoded by a sequence adjacent to and in an operon with *ahpC* (Antelmann et al., 1996). AhpA (encoded by *ykuU*), on the other hand, is a nonclassical Ahp and lacks this cognate AhpF (Broden et al., 2016). Instead, AhpA appears to use a thioredoxin‐like protein AhpT (encoded by *ykuV*) as its reductase (Broden et al., 2016; Rasmussen, Nielsen, & Jarmer, 2009; Zhang et al., 2006). In contrast to that seen for *ahpC*, we previously found that expression of *ahpA* is not altered upon mutation of the other peroxide‐scavenging enzymes (Broden et al., 2016). Furthermore, *ahpA* is not substantially expressed during exponential phase and does not appear to be required to protect cells from peroxides during vegetative growth (Broden et al., 2016). Instead, the expression of *ahpA* is regulated by the transition‐state regulatory protein AbrB (Broden et al., 2016; Chumsakul et al., 2011, 2013). AbrB primarily represses the expression of its target genes during exponential growth (Strauch & Hoch, 1993). However, the expression of *ahpA* is not substantially increased during stationary phase and *ahpA* mutants are not more sensitive to peroxides during stationary phase (Broden et al., 2016). Thus, additional factors may regulate expression of the *ahpA* gene. Given that a phenotype for an *ahpA* mutant has not yet been found, further investigation into when *ahpA* is expressed may help to elucidate its role in protection against oxidative stress (Broden et al., 2016).

The activity of AbrB is connected to that of Spo0A and the processes of sporulation and biofilm formation. Spo0A is a transcriptional regulator that controls the expression of numerous genes necessary for biofilm formation and sporulation (Fujita, González‐Pastor, & Losick, 2005; Molle et al., 2003). The activity of Spo0A is regulated through its phosphorylation; the concentration of Spo0A~P is determined by the activity of five different kinases, KinA‐E, via a phosphorelay system (Jiang, Shao, Perego, & Hoch, 2000; LeDeaux, Yu, & Grossman, 1995). Spo0A~P inhibits the expression of AbrB, and AbrB similarly indirectly inhibits the expression of Spo0A (Hamon & Lazazzera, 2001; Strauch, Webb, Spiegelman, & Hoch, 1990; Zuber & Losick, 1987). The concentration of Spo0A~P determines the gene expression profile in the cell, with intermediate levels of Spo0A~P leading to matrix gene expression and thus biofilm formation and high levels of Spo0A~P resulting in sporulation gene expression (Fujita et al., 2005).

*Bacillus subtilis* differentiates into spores under nutrient‐limiting conditions. Spores are metabolically dormant cells capable of surviving environmental stresses including desiccation and temperature extremes. Due at least in part to their structure and protein composition, dormant spores of *B. subtilis* are substantially more resistant than vegetative cells to H_2_O_2_ (Marquis, Sim, & Shin, 1994; Setlow & Setlow, 1993). Furthermore, spores produce a spore‐specific catalase, KatX, which is important in their resistance to H_2_O_2_ during germination (Bagyan et al., 1998).

Undomesticated strains of *B. subtilis* can form biofilms with complex architecture on either solid agar surfaces or on the surface of static liquid media (Branda, González‐Pastor, Ben‐Yehuda, Losick, & Kolter, 2001). Bacteria growing in biofilms are more resistant to disinfectants, including H_2_O_2_, than planktonic cells (Bridier, Briandet, Thomas, & Dubois‐Brissonnet, 2011; Sanchez‐Vizuete, Orgaz, Aymerich, Le Coq, & Briandet, 2015). This increased resistance is at least in part due to the multiple layers of cells and presence of the extracellular matrix inhibiting the penetration of cytotoxic compounds into the internal layers of the biofilm (Bridier et al., 2011). However, gene expression in biofilm‐grown cells differs drastically from that of planktonic cells, and thus altered gene expression may also contribute to the altered resistance of cells growing in biofilms (Bridier et al., 2011; Ren et al., 2004; Stanley, Britton, Grossman, & Lazazzera, 2003).

Given our previous studies demonstrating that the expression of *ahpA* is regulated by AbrB, the relationship of AbrB with Spo0A, and the increased resistance of both spores and biofilms to peroxides compared to vegetative cells, we decided to further explore the conditions under which *ahpA* is expressed. In these studies, we demonstrate that the expression of *ahpA* is not regulated in a manner similar to that of the other peroxide‐scavenging enzymes in *B. subtilis*. Instead, we demonstrate that the expression of *ahpA* is dependent on AbrB and Spo0A. We show that *ahpA* is not produced during the process of sporulation and that *ahpA* mutant strains are not defective in sporulation or germination. Instead, we show that *ahpA* is produced specifically during biofilm formation, establishing AhpA as a biofilm‐specific peroxidase for *B. subtilis*. These studies highlight the interplay between Spo0A and AbrB and suggest that derepression of *ahpA* by AbrB requires a signal or factor specific to biofilm formation conditions.

## 2 Materials and Methods

### 2.1 Bacterial strains and growth conditions

All *B. subtilis* strains, *Escherichia coli* strains, and plasmids used in this study are shown in Table S1. *Escherichia coli* strains were grown in LB medium. *Bacillus subtilis* strains were grown in LB medium, minimal medium, or MSgg biofilm medium at 30, 31, or 37°C. Broth cultures were incubated with shaking at 220 rpm. Details for each experiment are indicated in the figure legends. Minimal media contained 40 mmol/L potassium morpholinopropanesulfonic acid (MOPS; adjusted to pH 7.4 with KOH), 2 mmol/L potassium phosphate buffer (pH 7.0), glucose (2% wt/vol), (NH_4_)_2_SO_4_ (2 g/L), MgSO_4_·7H_2_0 (0.2 g/L), potassium glutamate (1 g/L), tryptophan (10 mg/L), 5 μmol/L FeCl_3_, and 80 nmol/L MnCl_2_ (Gabriel & Helmann, 2009). MSgg biofilm medium consisted of 5 mmol/L potassium phosphate (pH 7), 100 mmol/L MOPS (pH 7.4), 50 μmol/L FeCl_3_, 2 mmol/L MgCl_2_, 50 μmol/L MnCl_2_, 1 μmol/L ZnCl_2_, 700 μmol/L CaCl_2_, 2 μmol/L thiamine, 50 μg/ml tryptophan, 50 μg/ml phenylalanine, 50 μg/ml threonine, 0.5% glycerol, and 0.5% glutamate (Branda et al., 2001).

Routine molecular biology procedures were done as described previously (Sambrook & Russell, 2001). Isolation of *B. subtilis* chromosomal DNA, transformation, and specialized SP*β* transduction were done as described previously (Cutting & VanderHorn, 1990). Restriction enzymes (New England BioLabs), T4 DNA ligase (New England BioLabs), Phusion High‐Fidelity DNA polymerase (New England BioLabs), and Thermo‐Start master mix (Thermo Scientific) were used in accordance to the manufacturers' instructions.

### 2.2 *Bacillus subtilis* strain and plasmid construction

All primers used in this study for strain and plasmid construction are shown in Table S2. The *abrB*::tet^R^, *rsbW*::spc^R^, *sigF*::cam^R^, and *spoIIAB*::spc^R^ null mutations were made by long‐flanking homology PCR (Butcher & Helmann, 2006). Antibiotics were added as follows for selection: 1 μg/ml erythromycin and 25 μg/ml lincomycin (for macrolide‐lincosamide‐streptogramin B [MLS] resistance), 100 μg/ml spectinomycin, 8 μg/ml chloramphenicol, 10 μg/ml kanamycin, 5 μg/ml tetracycline, and 10 μg/ml neomycin.

*Escherichia coli* DH5α was used for routine DNA cloning as described previously (Sambrook & Russell, 2001). Ampicillin was used at a concentration of 100 μg/ml for selection of plasmids in *E. coli*. The P_spac_‐*kinA* allele was constructed by first cloning the *kinA* gene into vector pPL82 (Quisel, Burkholder, & Grossman, 2001). The *kinA* gene was amplified by PCR using primers P54 and P55. Both *kinA* and pPL82 were digested with the restriction enzymes *Hind*III and *Xba*I, ligated using T4 DNA ligase, and transformed into DH5α. The resulting plasmid was then linearized by digestion with PvuI and transformed into *B. subtilis*. The resulting construct was induced using 500 μmol/L isopropyl‐β‐___‐galactopyranoside (IPTG).

### 2.3 β‐galactosidase activity assays

Cells were grown in LB, minimal media, or MSgg biofilm media with aeration to an OD_600_ = 0.8 or for the indicated time period prior to analysis (Chai, Beauregard, Vlamakis, Losick, & Kolter, 2012; Chai, Chu, Kolter, & Losick, 2008). Details for each experiment are listed in the figure legends. All assays were performed in triplicate using independent cultures, and the values were averaged. β‐galactosidase activity was measured using a modification of the previously described method of Miller (Chen, James, & Helmann, 1993; Miller, 1972).

### 2.4 Induction of sporulation by the resuspension method

When measuring gene expression during sporulation, the process of sporulation was induced by the resuspension method, as described previously (Cutting & VanderHorn, 1990). Cells were grown in CH medium (Cutting & VanderHorn, 1990) at 37°C with aeration to an OD_600_ = 0.6. Cells were pelleted and resuspended in the same volume of Sterlini–Mandelstam (SM) resuspension medium and incubated at 37°C with aeration. Samples were taken at 0, 2, and 4 hr postresuspension to measure β‐galactosidase activity. All assays were performed in triplicate using independent cultures, and the values were averaged.

### 2.5 Sporulation efficiency assays

Efficiency of sporulation was determined by measuring the amount of heat‐resistant spores/ml (Ferguson, Camp, & Losick, 2007). Cells were grown in LB overnight and then diluted 1:100 into Difco sporulation medium (DSM) (Cutting & VanderHorn, 1990). DSM consisted of 8 g/L bacto‐nutrient broth, 0.1% (w/v) KCl, 0.012% (w/v) MgSO_4_·7H_2_O, 500 μmol/L NaOH, 1 mmol/L Ca(NO_3_)_2_, 10 μmol/L MnCl_2_, and 1 μmol/L FeSO_4_. Cells were grown with aeration at 37°C until reaching an OD_600_ = 0.6. At this time, 2 mmol/L H_2_O_2_ or 100 μmol/L CHP (diluted in ethanol) was added to each culture. Cultures were allowed to continue their incubation with aeration for a total of 48 hr. Vegetative cells were heat killed by incubating the cultures for 20 min at 80°C. The samples were then serial diluted and plated onto LB plates. After overnight growth at 37°C, resulting colonies were counted to determine the heat resistant spores/ml for each sample.

### 2.6 Germination assays

Germination assays were performed similarly to those described previously (Bagyan et al., 1998). Spores were prepared as described in Ferguson et al. (2007). Cells were induced to sporulate by growth in 100 ml DSM at 37°C with aeration for 48 hr. Spores were harvested by centrifugation and washed twice with distilled water. Next, the pellet was resuspended in 10 ml TE (10 mmol/L Tris, 1 mmol/L EDTA, pH 8.0) and 1 mg/ml lysozyme and incubated for 1 hr at 37°C with shaking. Sodium dodecyl sulfate was added (2 ml of 10% SDS) and the sample was further incubated for 20 min at 37°C. Spores were pelleted and washed twice in 0.01% Tween 20 and then two times with distilled water. Spores were stored at 4°C in water in the dark. All strains required for a single experiment were prepared together to ensure uniformity.

To measure germination and outgrowth, spores were first heated for 20 min at 80°C, and then cooled on ice. Spores were normalized to an OD_600_ = 1.0 in 10 mmol/L Tris‐HCl, pH 8.4, and then 675 μl spores were transferred to a 1.5 ml plastic cuvette and incubated at 37°C for 15 min. At time *t* = 0, 675 μl 2X concentrated LB and 150 μl 100 mmol/L l‐alanine (10 mmol/L final concentration) were added to the cuvette. Samples were incubated at 37°C throughout the germination and outgrowth assay, and samples were mixed with a pipette prior to each OD_600_ reading. 2 mmol/L H_2_O_2_ or 100 μmol/L CHP was added at *t* = 10 min. All experiments were done at least three times using at least two independent spore preparations. Shown in the figure is a representative experiment.

### 2.7 Biofilm colony assays

Biofilm colony assays were performed in a similar way to those described previously (Branda et al., 2001). Strains were grown on LB plates overnight at 30°C. Cells were scraped from the fresh LB plates and resuspended in LB to an OD_600_ = 1.0. Then, 2 μl of each cell suspension was spotted onto an MSgg plate and incubated for 72 hr at 30°C. All MSgg plates used in a given experiment were made at the same time. Plates were dried on the bench for 24 hr and then dried under a fume hood for an additional 15 min prior to use. Images of biofilms were captured using a Samsung ST201 16.1 mp digital camera mounted on a tripod.

### 2.8 Pellicle formation assays

Pellicle formation assays were performed as described previously (Branda et al., 2001; Yepes et al., 2012). Overnight cultures grown in LB at 31°C were diluted 1:100 into MSgg biofilm media. One mililiter of this diluted culture was then aliquoted into 24‐well polystyrene plates and incubated 24 hr at 30°C. H_2_O_2_ or CHP was added to each well at the concentration indicated in the figure. Peroxides were added either at the start of the 24 hr incubation or to 24‐hr‐old pellicles. Images of pellicles were captured using a Samsung ST201 16.1 mp digital camera.

## 3 Results

### 3.1 Expression of the *ahpA* gene is not controlled by regulatory proteins responsible for controlling the expression of other peroxide‐scavenging enzymes

Previous studies show that the expression of *ahpA* is low during vegetative growth (Broden et al., 2016; Nicolas et al., 2012; Rasmussen et al., 2009). This low expression level is likely in part due to the transition‐state regulator AbrB controlling the expression of *ahpA* (Broden et al., 2016; Chumsakul et al., 2011, 2013). However, in our previous studies, we were unable to detect a substantial increase in the expression of *ahpA* during stationary phase, and thus hypothesized that additional factors may influence the expression of the gene.

**Figure 1:**
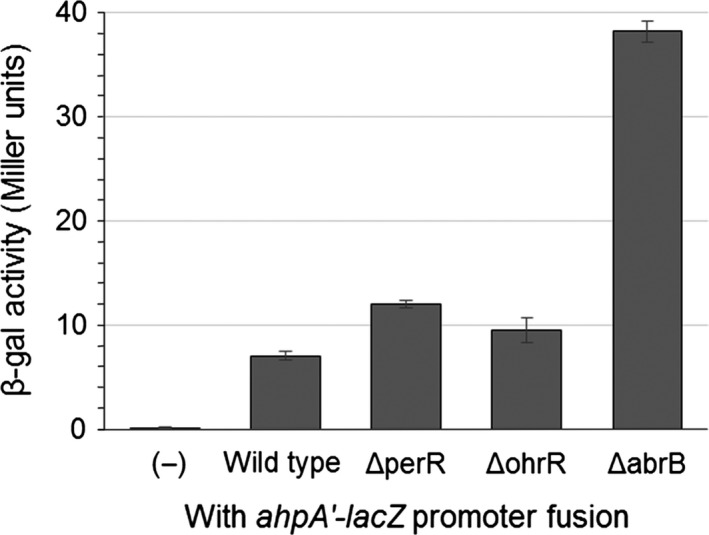
Expression of *ahpA* is not controlled by regulatory proteins responsible for controlling the expression of other peroxide‐scavenging enzymes. Shown are the expression levels of *ahpA* in strains lacking PerR, OhrR, or AbrB during vegetative growth based on β‐galactosidase activity from a transcriptional fusion of the *ahpA* promoter to *lacZ*. Strains were grown with aeration in LB broth at 37°C to an OD_600_ of 0.8. (−) represents a wild‐type strain lacking any *lacZ* fusion. Error bars indicate the standard error for three independent cultures

To further investigate the factors influencing the expression of *ahpA*, we first looked at the impact of regulatory proteins on *ahpA* expression that are known to control the expression of other peroxide‐scavenging enzymes in *B. subtilis*. To analyze the expression of *ahpA*, we used a promoter‐*lacZ* fusion containing the upstream region of *ahpA* linked to the *lacZ* gene and measured expression through the use of β‐galactosidase assays (Broden et al., 2016). Using this *lacZ* transcriptional fusion, we previously showed that the expression of *ahpA* is low during vegetative growth (<10 Miller units), but is substantially increased to about 38 Miller units upon mutation of *abrB* (Fig. 1) (Broden et al., 2016).

Both the primary vegetative catalase KatA and the classical alkylhydroperoxide reductase AhpC are regulated by the repressor protein PerR (Bsat, Herbig, Casillas‐Martinez, Setlow, & Helmann, 1998; Fuangthong et al., 2002). One of the organic hydroperoxide resistance proteins, OhrA, is also regulated by a repressor protein, OhrR (Fuangthong et al., 2001). Both PerR and OhrR are capable of sensing peroxides and respond to oxidative stress by derepressing their target genes (Fuangthong & Helmann, 2002; Lee & Helmann, 2006; Lee, Soonsanga, & Helmann, 2007). However, mutation of either *perR* or *ohrR* did not substantially increase the expression of *ahpA* (Fig. 1). Previous global transcriptional studies also did not identify *ahpA* as a member of the PerR regulon (Helmann et al., 2003). We note that since *perR* mutants grow very poorly, we used a faster growing *perR* suppressor strain for our analysis (Faulkner et al., 2012).

Consistent with these results, we found that exposure of cells to either hydrogen peroxide (H_2_O_2_), cumene hydroperoxide (CHP), or *t‐*butyl hydroperoxide (*t‐*BHP) did not increase the expression of *ahpA* (data not shown). Furthermore, we previously found that mutation of genes coding for peroxide‐scavenging enzymes did not substantially alter the expression of *ahpA*, supporting the idea that peroxide stress does not stimulate the expression of *ahpA* (Broden et al., 2016). Prior global transcriptional studies did not identify *ahpA* as a member of the H_2_O_2_ or *t‐*BHP stress stimulons (Helmann et al., 2003). While we did not test the influence of the disulfide‐stress transcriptional regulator Spx, which is responsible for regulating the expression of *tpx*, on the expression of *ahpA*, genome‐wide studies did not identify *ahpA* as a member of the Spx regulon (Nakano, Küster‐Schöck, Grossman, & Zuber, 2003; Rochat et al., 2012).

Three of the peroxide‐scavenging enzymes in *B. subtilis* are regulated by alternative sigma factors; the catalase KatE/B and the organic hydroperoxide resistance protein OhrB are regulated by the general stress sigma factor σ^B^, and a spore‐specific catalase KatX is regulated by an early forespore‐specific sporulation sigma factor σ^F^ (Bagyan et al., 1998; Engelmann, Lindner, & Hecker, 1995; Völker, Andersen, Antelmann, Devine, & Hecker, 1998). However, mutation of the genes coding for σ^B^, σ^F^, the antisigma factor for σ^B^ (*rsbW*), or the antisigma factor for σ^F^ (*spoIIAB*) did not alter the expression of *ahpA* (Fig. S1).

Taken all together, these results suggest that the expression of *ahpA* is not regulated by peroxide stress nor the regulatory proteins responsible for controlling the expression of the other peroxide‐scavenging enzymes in *B. subtilis*, making its regulation distinct from that of the other enzymes.

### 3.2 Expression of the *ahpA* gene is dependent on AbrB and Spo0A

**Figure 2:**
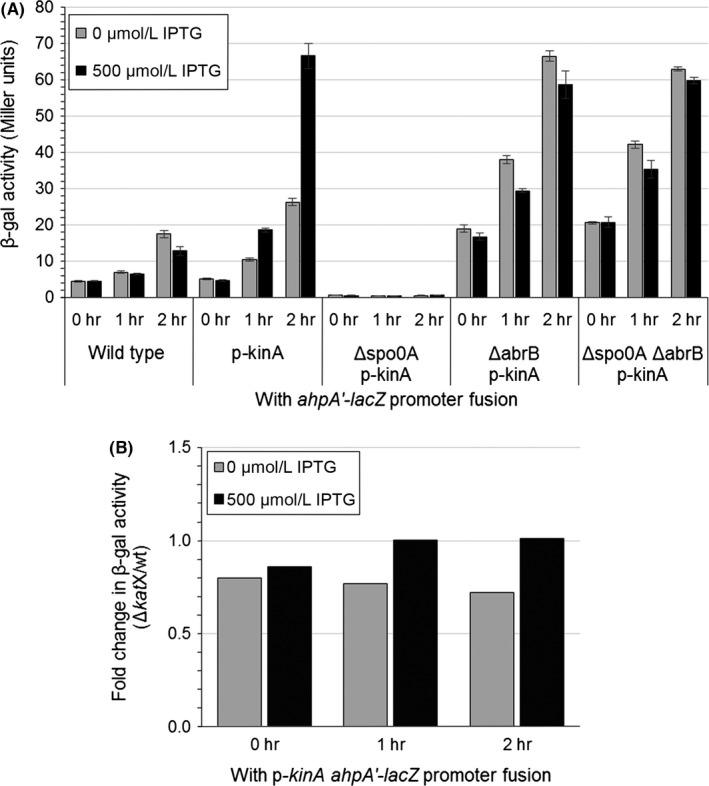
Phosphorylation of Spo0A increases the expression of *ahpA*. Shown are the expression levels of *ahpA* based on β‐galactosidase activity from a transcriptional fusion of the *ahpA* promoter to *lacZ*. The *kinA* gene was expressed from a P_spac_ promoter (p‐*kinA*). Strains were grown with aeration in LB broth at 37°C. At an OD_600_ of 0.4, 500 μmol/L IPTG was added to the indicated cultures (*t* = 0 hr). Samples were taken at 0, 1, and 2 hr postinduction. (A) Expression level of *ahpA* after induction of *kinA*. “Wild type” represents a strain containing the *lacZ* fusion but lacking the p‐*kinA* construct. Error bars indicate the standard error for three independent cultures. (B) Fold change in β‐galactosidase activity from the *ahpA'‐lacZ* fusion resulting from the induction of *kinA* in strains lacking *katX* compared to isogenic strains containing wild‐type *katX* (Δ*katX*/wild type)

Since the activity of AbrB is connected to that of Spo0A and the processes of sporulation and biofilm formation, we next looked to determine if Spo0A impacts the expression of *ahpA*. We cloned *kinA* into an IPTG‐inducible plasmid (pPL82) and integrated this plasmid into the *B. subtilis* chromosome. Expression of *kinA* should result in the phosphorylation, and thus activation, of Spo0A (Burbulys, Trach, & Hoch, 1991; Eswaramoorthy et al., 2010). We found that expression of *kinA* substantially increased the expression of *ahpA* via our *ahpA'‐lacZ* fusion from around 20 Miller units when *kinA* was not induced to over 60 Miller units when *kinA* was induced (Fig. 2A). Expression of *ahpA* was dependent on Spo0A when wild‐type AbrB was present. However, in the absence of AbrB, the expression of *ahpA* was no longer dependent on the presence or phosphorylation of Spo0A. These results demonstrate that the expression of *ahpA* is regulated by AbrB and suggest that Spo0A influences the expression of *ahpA* through its inhibition of *abrB*.

### 3.3 The *ahpA* gene is not produced during sporulation

**Figure 3:**
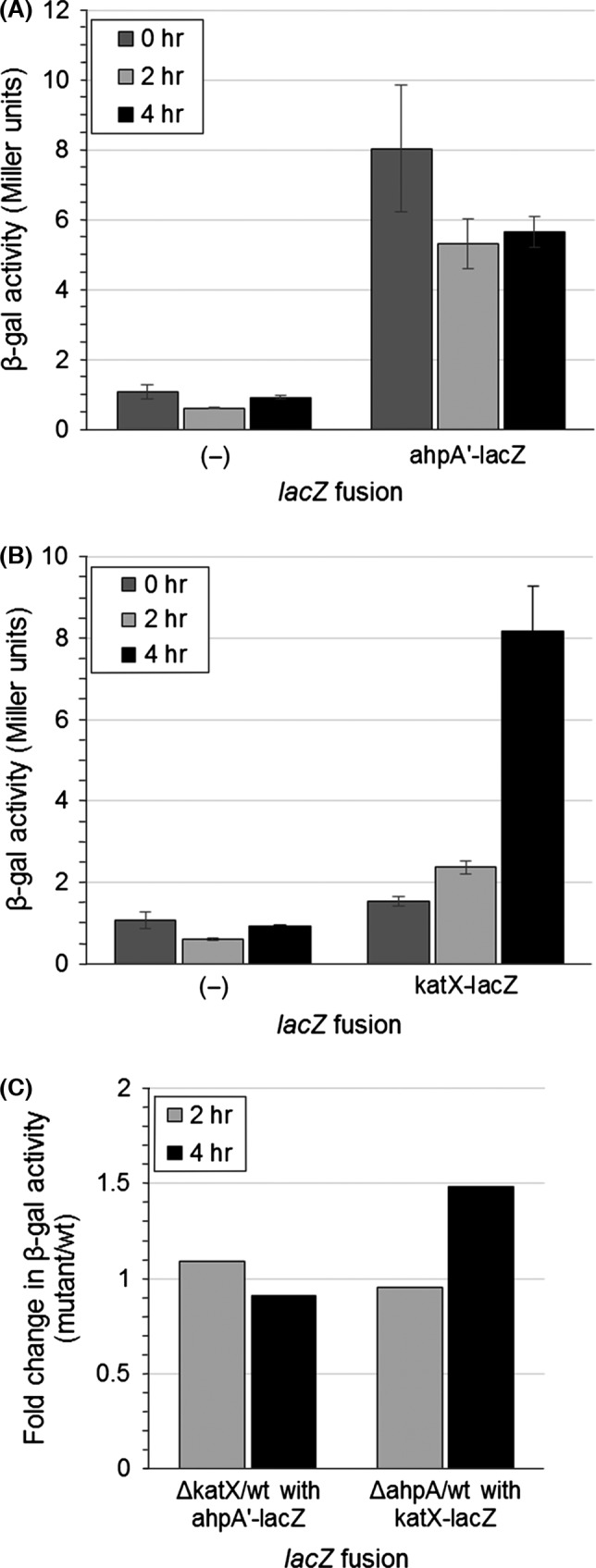
The *ahpA* gene is not expressed during sporulation. Sporulation was induced by the resuspension method, and samples were taken 0, 2, or 4 hr postresuspension. Shown are the expression levels of *ahpA* (A) and *katX* (B) based on β‐galactosidase activity from a fusion of the *ahpA* promoter or *katX* to *lacZ*. Error bars indicate the standard error for three independent cultures. (C) Fold change in expression of *ahpA* (left) or *katX* (right) in strains lacking *katX* (left) or *ahpA* (right) compared to isogenic wild‐type strains (mutant/wild type)

Given that there is a spore‐specific catalase, KatX, we looked at the potential role for AhpA during sporulation (Bagyan et al., 1998). Using the resuspension method, we induced the process of sporulation in strains containing our *ahpA'‐lacZ* fusion. Even after 4 hr postinduction of sporulation, we did not see an increase in the expression of *ahpA* from the *ahpA'‐lacZ* fusion (Fig. 3A). In contrast, by 4 hr we did see an approximately fivefold increase in the expression of *katX* (Fig. 3B). Thus, it does not appear that *ahpA* is produced during sporulation. This is consistent with our previous observation that *ahpA* is not regulated by the same forespore‐specific sporulation sigma factor σ^F^ responsible for controlling the expression of *katX* (Fig. S1) (Bagyan et al., 1998).

Mutation of either *ahpC* or *katA* results in the increased expression of other proteins responsible for protecting cells against peroxides (Antelmann et al., 1996; Bsat et al., 1996; Chen et al., 1995). We reasoned that there might be a similar cooperative relationship between *katX* and *ahpA*. However, we found that *ahpA* expression was basically unchanged in *katX* mutants compared to wild type (<1.5‐fold change) (Fig. 2B, 3C). Similarly, expression of *katX* was not substantially changed in *ahpA* mutants compared to wild type (<1.5‐fold change) (Fig. 3C). Given that the expression of *ahpA* does not change in response to peroxide stress, this lack of coordination between the expression of *katX* and *ahpA* is not surprising.

### 3.4 AhpA is not required to protect spores from peroxides

**Figure 4:**
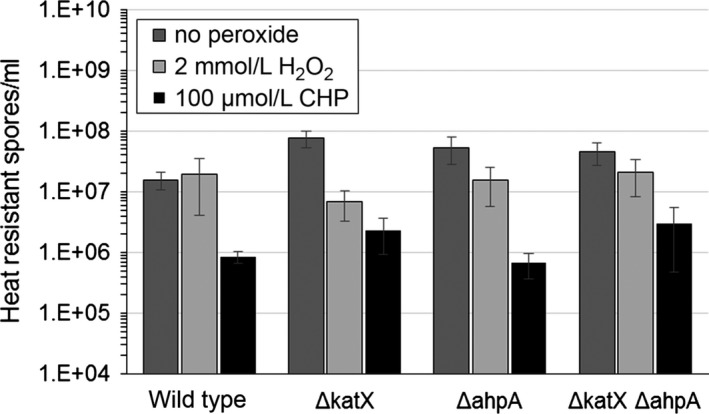
Peroxides do not decreases the sporulation efficiency of *ahpA* and *katX* mutant strains, as measured by the amount of heat resistance spores/ml produced. Strains were grown in DSM with aeration for 48 hr to induce sporulation. Either 2 mmol/L H_2_O_2_ or 100 μmol/L cumene hydroperoxide (CHP) was added to the DSM culture when the OD_600_ = 0.6. Error bars indicate the standard error for either six (no peroxide), four (H_2_O_2_), or two (CHP) independent cultures. *p* < .05 using Student's *t* test for almost all samples when compared to the wild‐type strain for the same peroxide treatment, except *p* = .03 for the Δ*katX* strain with no peroxide compared to the wild‐type sample with no peroxide

Previous studies showed that already formed spores lacking *ahpA* or both *ahpA* and *ahpC* were not any more sensitive to either CHP or *t*‐BHP than wild type (Casillas‐Martinez & Setlow, 1997). Spores lacking *katX* were also not any more sensitive to H_2_O_2_ than wild type (Casillas‐Martinez & Setlow, 1997). We looked at the efficiency of sporulation of strains containing mutations in *ahpA* and/or *katX*. We exposed strains to either 2 mmol/L H_2_O_2_ or 100 μmol/L CHP during the process of sporulation in DSM and analyzed the resulting amount of heat‐resistant spores/ml produced by each strain. In the absence of peroxides, we did not detect any decrease in spore formation in either the *ahpA* single mutant or the *katX ahpA* double mutant compared to wild type (Fig. 4). Addition of either 2 mmol/L H_2_O_2_ or 100 μmol/L CHP during the process of spore formation also did not substantially decrease the amount of spores formed for any of the strains, as there was a less than 10‐fold change in the amount of spores produced by each of these strains compared to wild type exposed to the same peroxide. Thus, it does not appear that *ahpA* is needed by cells during sporulation. Previous studies also showed that *katX* mutant strains were not any more sensitive to H_2_O_2_ during sporulation than wild type (Bagyan et al., 1998).

**Figure 5:**
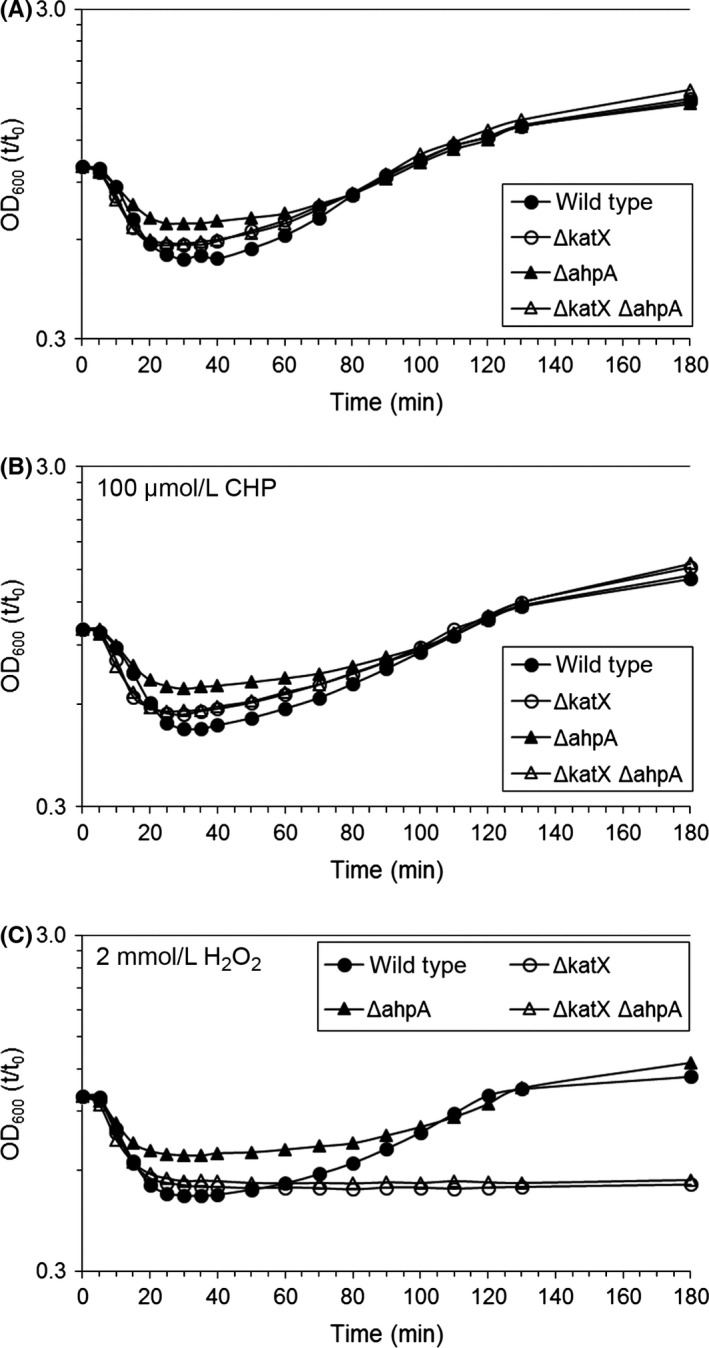
Peroxides do not impair the outgrowth of spores containing mutations in *ahpA*. The germination and outgrowth of spores was measured by monitoring the OD_600_ of the samples. Either no peroxide (A), 100 μmol/L cumene hydroperoxide (CHP) (B), or 2 mmol/L H_2_O_2_ (C) was added 10 min postinduction of germination. Shown is the ratio of the optical density at each time point compared to *t* = 0 (*t*/*t*_0_). All experiments were done at least three times using at least two independent spore preparations. All three panels show the results from a single spore preparation

Strains lacking *katX* were previously shown to be sensitive to 2 mmol/L H_2_O_2_ during spore outgrowth (Bagyan et al., 1998). Thus, we analyzed the germination and outgrowth of spores containing mutations in *ahpA* and/or *katX* by following the OD_600_ of the sample after onset of germination. We found that spores lacking either *katX* or both *katX* and *ahpA* were impaired in their outgrowth upon exposure to 2 mmol/L H_2_O_2_. However, mutation of *ahpA* had no impact on the outgrowth of spores, even when exposed to either 100 μmol/L CHP or 2 mmol/L H_2_O_2_ during germination (Fig. 5). Although the results shown in Fig. 5 show a slightly smaller drop in the OD_600_ of the *ahpA* mutant spores compared to wild type, we did not see this difference in OD_600_ when analyzing other spore preparations. Taking all of the data surrounding the process of sporulation in account, it does not appear that AhpA is produced in spores or required by spores for protection against peroxides.

### 3.5 The *ahpA* gene is expressed during biofilm formation

Previous analysis of the Spo0A regulon did not identify *ahpA* as a member of the regulon (Molle et al., 2003). However, *ahpA* was identified as a “low‐threshold” gene activated by low levels of Spo0A~P (9.8‐fold change in transcript level compared to cells lacking *spo0A*) (Fujita et al., 2005). These prior studies and results in Fig. 2 suggest that *ahpA* may be produced during biofilm formation. The wild‐type strain (CU1065, a 168 derivative) used in the studies previously described in this report is a domesticated laboratory strain and does not form robust biofilms (Branda et al., 2001). Therefore, we moved our *lacZ* fusions and null mutations into an undomesticated strain background, 3610, for our analysis of the role of *ahpA* in biofilms.

**Figure 6:**
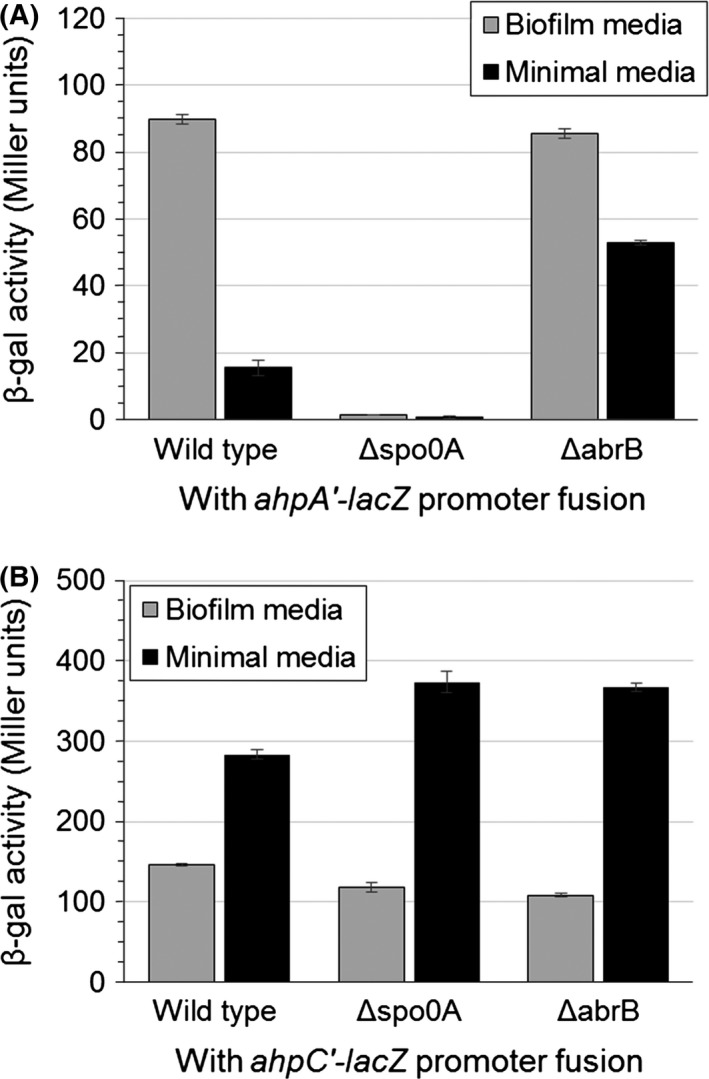
Expression of *ahpA* is increased during biofilm formation. Shown are the expression levels of *ahpA* (A) and *ahpC* (B) based on β‐galactosidase activity from a fusion of the *ahpA* or *ahpC* promoter to *lacZ*. Strains were grown for 24 hr at 31°C in borosilicate glass culture tubes with aeration in either biofilm‐promoting media (MSgg) or minimal media. Error bars indicate the standard error for three independent cultures

To analyze gene expression in biofilms, we compared the expression of *ahpA* from our *ahpA'‐lacZ* fusion in 24‐hr cultures grown at 31°C in either minimal media (nonbiofilm conditions) or MSgg biofilm‐promoting media (Chai et al., 2008, 2012). We found that the expression of *ahpA* was increased sixfold in the MSgg biofilm media, measuring at 90 Miller units compared to 16 Miller units in nonbiofilm conditions (Fig. 6A). This expression in MSgg was dependent on Spo0A. A strain lacking *abrB* showed a similar level of expression of *ahpA* as wild type when grown in MSgg. In contrast, the *abrB* mutant showed an increase in expression of *ahpA* compared to wild type (53 vs. 16 Miller units) when grown in nonbiofilm conditions. Thus, our results show that *ahpA* is expressed during biofilm formation, but not stationary phase, through the actions of AbrB and Spo0A. These results furthermore suggest that despite the expression of *ahpA* being controlled by the transition‐state regulator AbrB, entry into stationary phase is not sufficient to initiate the derepression of *ahpA*. Our results suggest that an additional signal or factor present during biofilm formation is required for the expression of *ahpA*, with Spo0A possibly having a roll in initiating this signal. When we followed the expression of *ahpA* through exponential phase to stationary phase, we did not detect any substantial increase in its expression after 6 hr of growth in either MSgg or minimal media at 31°C, nor did we detect any substantial differences in expression between the two media types at any given time point during this 6 hr (Fig. S2).

This expression of *ahpA* in biofilms is consistent with previous genome‐wide studies that identified *ahpA* being expressed during biofilm formation (Ren et al., 2004; Stanley et al., 2003). Stanley et al. (2003) showed a 2.6‐fold increase in 24‐hr‐old biofilms. Similarly, Ren et al. (2004) showed a 3.1‐fold change in *ahpA* expression in 5‐day‐old biofilms. Furthermore, Ren et al. showed that this change in expression of *ahpA* was not dependent on SpoIIGB, indicating that the expression of *ahpA* was independent of sporulation.

We also analyzed the expression of the gene encoding the classical Ahp, *ahpC*, during biofilm formation (Fig. 6B). We detected a high level of *ahpC* expression when cells were grown in either MSgg (146 Miller units) or minimal media (283 Miller units). Previous studies show that the expression of both *katA* and *ahpC* was not found to be increased in biofilms compared to planktonic cells (Fujita et al., 2005; Ren et al., 2004; Stanley et al., 2003). Furthermore, both *ahpC* and *katA* are expressed in stationary phase cells, but *ahpA* is not (Bol & Yasbin, 1994; Broden et al., 2016; Bsat et al., 1996; Engelmann et al., 1995). Thus, only *ahpA* appears to be biofilm specific.

### 3.6 Strains containing a null mutation in *ahpA* can still form robust biofilms

**Figure 7:**
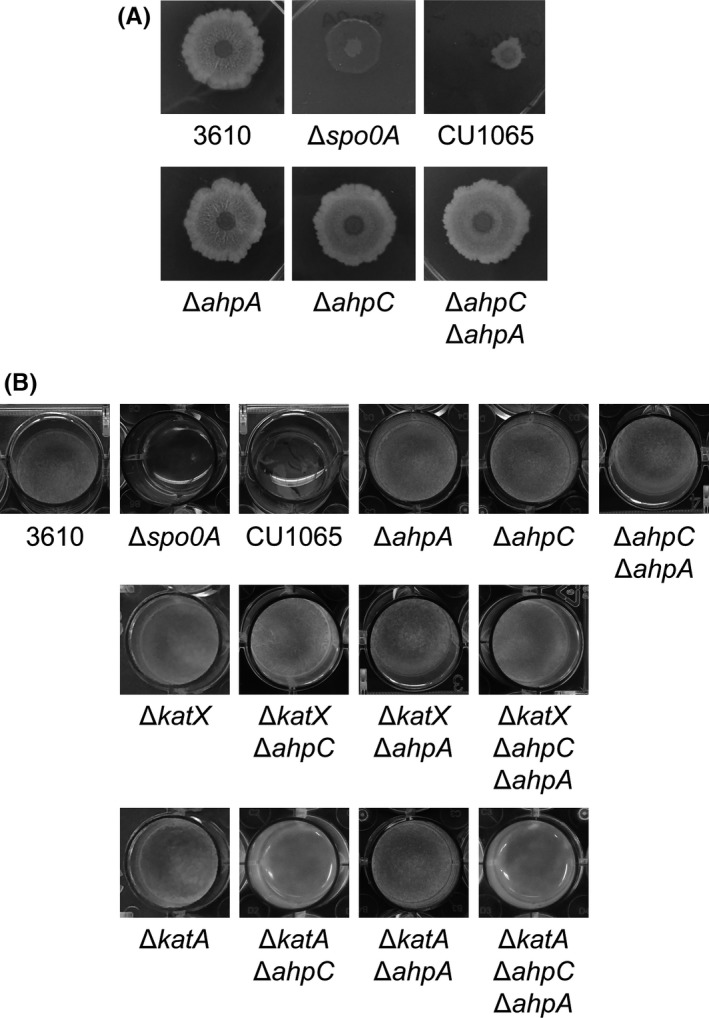
Mutation of *ahpA* does not impair biofilm formation. Biofilm formation was observed by colony morphology (A) or pellicle assay (B). (A) 2 μl of a cell suspension of each strain was spotted onto an MSgg agar plate and incubated for 72 hr at 30°C. 3610 wild‐type biofilms measured about 18–20 mm in diameter. (B) Cultures were grown in MSgg broth media in 24‐well polystyrene plates for 24 hr at 30°C. 3610, undomesticated (biofilm forming) wild‐type strain (strain DK1042). CU1065, domesticated (nonbiofilm forming) wild‐type strain. All mutants shown are in the 3610 (DK1042) background strain

We next assessed the effect of the *ahpA* mutation on biofilm formation. We observed biofilm formation in colony assays on MSgg agar plates grown at 30°C for 72 hr. We found that *ahpA* mutants formed similarly looking biofilms as 3610 wild type (Fig. 7A). Interestingly, the *ahpC* mutant strain did form slightly flatter colonies than wild type, but the effect was very small and not visible in pellicle assays (Fig. 7A and B). The *ahpC ahpA* double mutant formed colony biofilms similar to those of the *ahpC* single mutant.

We also grew the strains as pellicles in 24‐well plates containing MSgg at 30°C for 24 hr. Again, we did not detect any defect in pellicle formation by the *ahpA* mutant strain (Fig. 7B). We also did not detect any defects in pellicle formation in mutants lacking both *ahpA* and one or two additional peroxide‐scavenging enzymes (*ahpC*,* katA*, or *katX*). The *katA ahpC* and *katA ahpC ahpA* mutant strains grew in MSgg but did not form pellicles; there did not appear to be a substantial difference in growth or pellicle formation between the two strains. This lack of pellicle formation is likely related to the poor growth of these two strains in minimal media (Broden et al., 2016; Bsat et al., 1996).

**Figure 8:**
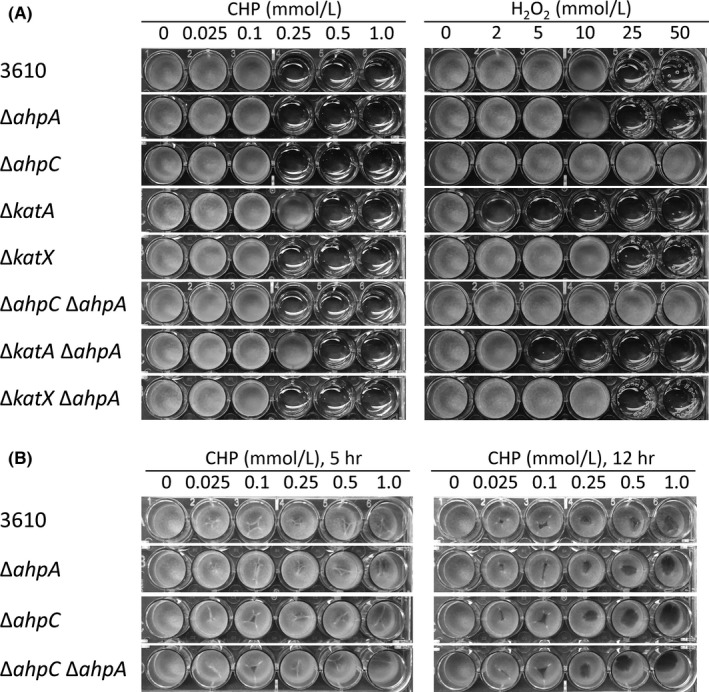
Mutation of *ahpA* does not make pellicles more susceptible to peroxides. Cultures were grown in MSgg broth media in 24‐well polystyrene plates for 24 hr at 30°C. 3610, undomesticated wild‐type strain (strain DK1042). (A) Cumene hydroperoxide (CHP) or H_2_O_2_ was added when the cultures were first inoculated into MSgg. (B) CHP was added after pellicles were grown for 24 hr and further incubated for the indicated time

We next grew the pellicles in the presence of either CHP or H_2_O_2_. Wild‐type 3610 formed pellicles at 100 μmol/L CHP and 10 mmol/L H_2_O_2_. However, mutation of *ahpA*, either alone or in combination with a mutation in either *ahpC*,* katX*, or *katA*, did not impair pellicle formation (Fig. 8A). The *katA* mutant strains were slightly more resistant to CHP than wild type, forming pellicles at 250 μmol/L CHP, consistent with this mutation increasing the expression of other oxidative stress genes (Chen et al., 1995) (Fig. 8A). Similarly, the *ahpC* mutant strains were more resistant to H_2_O_2_ than wild type, forming pellicles at 50 mmol/L H_2_O_2_ (Fig. 8A) (Antelmann et al., 1996; Bsat et al., 1996; Fuangthong et al., 2001). Strains containing *katA* mutations were sensitive to H_2_O_2_ (Bol & Yasbin, 1991) (Fig. 8A).

We additionally exposed 24‐hr‐old pellicles to CHP or H_2_O_2_ to assess if *ahpA* is required to maintain already formed biofilms. Pellicles exposed to 250 μmol/L CHP began to show damage 5 hr postperoxide exposure and showed obvious damage by 12 hr; however, mutation of *ahpA* had no effect on the degree or amount of CHP required for this damage (Fig. 8B). Similarly, mutation of *ahpA* alone or in combination with mutation of *ahpC*,* katX*, or *katA* had no effect on the degree or amount of H_2_O_2_ required to damage established pellicles (data not shown). Taken together, it appears that mutation of *ahpA* does not impair biofilm formation, even in the presence of peroxides.

## 4 Discussion

The results presented in this study provide insight into when during growth AhpA is produced by *B. subtilis*. We and others have shown that the transition‐state regulator AbrB controls, most likely directly, the expression of *ahpA* (Broden et al., 2016; Chumsakul et al., 2011, 2013). However, in prior studies, we were unable to detect any substantial increase in the expression of *ahpA* during stationary phase, suggesting that additional factors may influence the expression of the gene (Broden et al., 2016). The studies presented here show that both the phosphorylation of Spo0A and the induction of biofilm formation stimulate the expression of *ahpA* in a Spo0A‐ and AbrB‐dependent manner. The *ahpA* gene is not expressed to a substantial level during vegetative growth, stationary phase, or sporulation, nor does its expression appear to be increased during oxidative stress (Broden et al., 2016). Therefore, based on our current evidence, it appears that AhpA is a biofilm‐specific peroxidase.

These studies highlight how the interplay between Spo0A and AbrB helps to fine tune the regulatory mechanism of AbrB. Although AbrB is known to allow expression of its target genes in stationary phase, in this case an additional signal or factor present in biofilms is needed for derepression. One possible factor may be that of the antirepressor AbbA. AbbA is a member of the Spo0A regulon that aids in the removal of AbrB from its target DNA, promoting derepression of the target gene (Banse, Chastanet, Rahn‐Lee, Hobbs, & Losick, 2008; Molle et al., 2003; Tucker et al., 2014). It is possible that the level of Spo0A~P required for induction of AbbA may not be reached until biofilm‐promoting conditions arise. A second possible signal may be the phosphorylation of serine 86 of AbrB, which inhibits the binding of the protein to DNA (Kobir et al., 2014). Three serine/threonine kinases (PrkC, PrkD, and YabT) contribute to this inactivation of AbrB (Kobir et al., 2014). Of these three kinases, one in particular, PrkC is expressed in biofilms and required for efficient biofilm formation (Madec, Laszkiewicz, Iwanicki, Obuchowski, & Séror, 2002). Therefore, it is possible that expression of PrkC during biofilm formation may promote the phosphorylation of AbrB bound to the *ahpA* promoter. Additional studies are required to demonstrate if one or both of these mechanisms are required for the derepression of *ahpA* by AbrB. Alternatively, Spo0A~P may induce the expression of an unknown regulatory protein that contributes to the expression of *ahpA*. However, since the level of *ahpA* expression is comparable among the *abrB* mutant strain and the other induction conditions we tested, it appears that AbrB is the primary regulatory protein responsible for controlling the expression of *ahpA*.

Although we detect substantial expression of *ahpA* during biofilm formation, an *ahpA* mutant still forms robust biofilms. However, there is often overlap in the activity of the peroxide‐scavenging enzymes, and thus we may have not identified the precise combination of mutations required to reveal a sensitive phenotype for the *ahpA* mutant. Additionally, while previous studies demonstrate that AhpA is capable of scavenging peroxides, we do not yet know if this is in fact its in vivo activity (Broden et al., 2016; Cha, Bae, Kim, Park, & Kim, 2015). Therefore, additional studies are needed to both determine the preferred substrate of AhpA and the role of AhpA in protecting biofilms from oxidative stress.

Although AhpC and KatA do not appear to be biofilm‐specific, their expression also does not appear to be substantially repressed in biofilms compared to planktonic cells (Fujita et al., 2005; Ren et al., 2004; Stanley et al., 2003). They are also both expressed well in stationary phase cells (Bol & Yasbin, 1994; Bsat et al., 1996; Engelmann et al., 1995). The studies presented here demonstrate that *ahpC* is expressed well in both biofilms and stationary phase planktonic cells and that strains lacking both *ahpC* and *katA* do not form robust pellicles. Therefore, AhpC and KatA appear to contribute to the protection of biofilms against peroxides, but not in the specific manner that may be the case for AhpA.

Bacteria growing in biofilms are known to be more tolerant to various biocides including H_2_O_2_ (Bridier et al., 2011; Sanchez‐Vizuete et al., 2015). While some of this tolerance is attributed to the three‐dimensional architecture of the biofilm and its extracellular matrix, part of the tolerance may also be due to altered expression of stress‐resistance genes (Bridier et al., 2011). Indeed, although *Pseudomonas aeruginosa* strains containing mutations in the catalase gene *katA* are able to form robust biofilms, they are unable to maintain their biofilm structural integrity upon exposure to H_2_O_2_ (Elkins, Hassett, Stewart, Schweizer, & McDermott, 1999). The tolerance of the wild‐type biofilms to H_2_O_2_ was attributed to the ability of catalase to neutralize the peroxide, preventing its penetration into the bottom layers of the biofilm (Shin, Choi, & Cho, 2008; Stewart et al., 2000).

*Campylobacter jejuni* AhpC was also shown to influence biofilm formation (Oh & Jeon, 2014). Studies show that biofilm formation was enhanced in *C. jejuni ahpC* mutant strains and decreased in strains overexpressing *ahpC*. The enhanced biofilm formation was attributed to the accumulation of reactive oxygen species in the *ahpC* mutant (Oh & Jeon, 2014). Interestingly, *C. jejuni* AhpC does not have a cognate AhpF, and thus is a nonclassical Ahp, similar to AhpA (Baillon, Van Vliet, Ketley, Constantinidou, & Penn, 1999; Broden et al., 2016).

Overall, these studies show that AhpA from *B. subtilis* has a distinct role from that of its homolog AhpC during growth of the organism. These studies demonstrate that *ahpA* is specifically expressed during biofilm formation and that this expression depends on AbrB and Spo0A. This observation parallels that of the three catalases produced in *B. subtilis*. The major vegetative catalase in *B. subtilis*, KatA, is expressed under conditions of oxidative stress through the derepression of PerR; KatE is regulated as part of the σ^B^ general stress response; and KatX is important in germinating spores (Bagyan et al., 1998; Engelmann & Hecker, 1996). Thus, the three catalases are used by the cell under different growth conditions, with one that is specific to spores. Similarly, the two Ahp peroxidases appear to be used under different growth conditions, with one that is specific to biofilms. The results presented here combined with previous studies on other nonclassical (non‐AhpF utilizing) Ahp proteins lead us to hypothesize that they often have roles distinct from their use during vegetative growth; however, further studies on additional nonclassical Ahp proteins are required to determine if this is indeed the case.

## Funding Information

Beta Beta Beta Research Scholarship Foundation Fund provided funding to Joelie Zwick, Sarah Noble, and Alyssa N. King. Bradley University provided funding to Joelie Zwick, Sarah Noble, Alyssa N. King, and Melinda J. Faulkner.

## Conflict of Interest

None declared.
