# Supplementary material for: AhpA is a peroxidase expressed during biofilm formation in Bacillus subtilis
Source: Microbiologyopen. 2016 Sep 28;6(1):e00403. doi: 10.1002/mbo3.403 (PMC5300871; doi:10.1002/mbo3.403)
Supplement: Supplementary file 1 [file MBO3-6-0-s001.docx]

**AhpA is a peroxidase expressed during biofilm formation in *Bacillus subtilis***

Joelie V. Zwick, Sarah Noble, Yasser K. Ellaicy, Gabrielle Dierker Coe, Dylan J. Hakey, Alyssa N. King, Alex J. Sadauskas, and Melinda J. Faulkner^*^

**SUPPLEMENTAL TABLES**

**Table S1.** Strains and plasmids used in this study

|  | **Strain or Plasmid** | **Genotype** | **Reference** |
| --- | --- | --- | --- |
| *B. subtilis* strains | | | |
|  | CU1065 | W168 *att*SPβ *trpC*2 | Lab collection |
|  | DK1042 | 3610 *comI*(Q12L) | (Konkol *et al.*, 2013) |
|  | IB447 | PS832 *katA*::erm *katX::katX-lacZ* (cam^R^) | (Bagyan *et al.*, 1998) |
|  | PS2664 | PS832 *katA:*:cam^R^ *katX*::erm^R^ | (Bagyan *et al.*, 1998) |
|  | RL2242 | PY79 *spo0A*::spc^R^ | (Fawcett *et al.*, 2000) |
|  | HB14075 | CU1065 Δ*perR*::spc^R^ supp2.1 | (Faulkner *et al.*, 2012) |
|  | HB2000 | CU1065 Δ*ohrR*::kan^R^ | (Fuangthong *et al.*, 2001) |
|  | HB355 | CU1065 Δ*abrB*::cam^R^ | Helmann lab |
|  | FB145 | CU1065 Δ*abrB*::tet^R^ | This study |
|  | FB146 | CU1065 Δ*spo0A*::spc^R^ | This study |
|  | FB147 | CU1065 Δ*spo0A*::spc^R^ Δ*abrB*::tet^R^ | This study |
|  | HB553 | CU1065 Δ*sigB*::cam^R^ | Helmann lab |
|  | FB077 | CU1065 Δ*rsbW*::spc^R^ | This study |
|  | FB081 | CU1065 Δ*sigB*::cam^R^ Δ*rsbW*::spc^R^ SPβ*c*2Δ2::Tn*917*::φ(*ahpA*’-*cat*-*lacZ*) | This study |
|  | FB087 | CU1065 Δ*sigF*::cam^R^ | This study |
|  | FB089 | CU1065 Δ*spoIIAB*::spc^R^ | This study |
|  | FB091 | CU1065 Δ*sigF*::cam^R^ Δ*spoIIAB*::spc^R^ | This study |
|  | FB004 | CU1065 Δ*ahpA*::spc^R^ | (Broden *et al.*, 2016) |
|  | FB001 | CU1065 Δ*ahpCF*::tet^R^ | (Broden *et al.*, 2016) |
|  | FB078 | CU1065 Δ*katX*::erm^R^ | This study |
|  | FB073 | CU1065 Δ*katX*::erm^R^ Δ*ahpA*::spc^R^ | This study |
|  | FB016 | CU1065 SPβ*c*2Δ2::Tn*917*::φ(*ahpA*’-*cat*-*lacZ*) | (Broden *et al.*, 2016) |
|  | HB2083 | CU1065 SPβ*c*2Δ2::Tn*917*::φ(*ahpC*’-*cat*-*lacZ*) | (Fuangthong *et al.*, 2002) |
|  | FB083 | CU1065 *katX::katX-lacZ* (cam^R^) | This study |
|  | FB085 | CU1065 *amyE*::P_spac_-*kinA* | This study |
|  | FB123 | DK1042 Δ*spo0A*::spc^R^ | This study |
|  | FB138 | DK1042 Δ*abrB*::cam^R^ | This study |
|  | FB149 | DK1042 Δ*abrB*::tet^R^ | This study |
|  | FB121 | DK1042 Δ*ahpA*::spc^R^ | This study |
|  | FB120 | DK1042 Δ*ahpCF*::tet^R^ | This study |
|  | FB132 | DK1042 Δ*katA*::mls^R^ | This study |
|  | FB131 | DK1042 Δ*katX*::erm^R^ | This study |
|  | FB124 | DK1042 Δ*ahpC*::tet^R^ Δ*ahpA*::spc^R^ | This study |
|  | FB129 | DK1042 Δ*katA*::mls^R^ Δ*ahpA*::spc^R^ | This study |
|  | FB130 | DK1042 Δ*katX*::erm^R^ Δ*ahpA*::spc^R^ | This study |
|  | FB133 | DK1042 Δ*katX*::erm^R^ Δ*ahpC*::tet^R^ | This study |
|  | FB134 | DK1042 Δ*katA*::mls^R^ Δ*ahpC*::tet^R^ | This study |
|  | FB135 | DK1042 Δ*katX*::erm^R^ Δ*ahpC*::tet^R^ Δ*ahpA*::spc^R^ | This study |
|  | FB136 | DK1042 Δ*katA*::mls^R^ Δ*ahpC*::tet^R^ Δ*ahpA*::spc^R^ | This study |
|  | FB137 | DK1042 SPβ*c*2Δ2::Tn*917*::φ(*ahpA*’-*cat*-*lacZ*) | This study |
|  | FB126 | DK1042 SPβ*c*2Δ2::Tn*917*::φ(*ahpC*’-*cat*-*lacZ*) | This study |
| *B. subtilis* plasmids | | | |
|  | pPL82 | amp^R^, cam^R^, P_spac_ promoter, IPTG inducible, inserts at *amyE* site | (Quisel *et al.*, 2001) |
|  |  |  |  |
| *E. coli* strains | | | |
|  | DH5α | *fhuA2 lac(Δ)U169 phoA glnV44 Φ80' lacZ(Δ)M15 gyrA96 recA1 relA1 endA1 thi-1 hsdR17* | Lab collection |

Cam, chloramphenicol; erm, erythromycin; spc, spectinomycin; kan, kanamycin; tet, tetracycline; mls, macrolide-lincosamide-streptogramin B

**Table S2.** Primers used in this study

| **Primer** | **Allele** | **Primer sequence^1^** |
| --- | --- | --- |
| P50 | Δ*rsbW*::spc^R^ | CGATTTAGAGGCCAAAGGACTCG |
| P51 | Δ*rsbW*::spc^R^ | CGTTACGTTATTAGCGAGCCAGTCCGGCACTTTCATTTCGATGTAATC |
| P52 | Δ*rsbW*::spc^R^ | CAATAAACCCTTGCCCTCGCTACGCGAGTTGATCATGACACAACCATC |
| P53 | Δ*rsbW*::spc^R^ | CGTGCATTTGAGATATACCGAGAATG |
| P54 | P_spac_-*kinA* | GTCGACAAGCTTGTCGAAACACGATGATCATGC |
| P55 | P_spac_-*kinA* | GTCGACTCTAGAGCAATGCAGACGATAAACAACG |
| P56 | Δ*sigF*::cam^R^ | GACTCAATCATTGGAGAAGGATG |
| P57 | Δ*sigF*::cam^R^ | CTTGATAATAAGGGTAACTATTGCCCACATCCATAACAAATCTCCTTAATTAC |
| P58 | Δ*sigF*::cam^R^ | GGGTAACTAGCCTCGCCGGTCCACGCAAGGTTCAAATGGATCATACG |
| P59 | Δ*sigF*::cam^R^ | GCCACATATTGATCGAGATCAAG |
| P60 | Δ*spoIIAB*::spc^R^ | GCGAGGATATGAACGATGTG |
| P61 | Δ*spoIIAB*::spc^R^ | CGTTACGTTATTAGCGAGCCAGTCGTGCATTTCATTTTTCATGATGC |
| P62 | Δ*spoIIAB*::spc^R^ | CAATAAACCCTTGCCCTCGCTACGGCGCTTTGTAATTAAGGAGATTTG |
| P63 | Δ*spoIIAB*::spc^R^ | GACTAGCCATCCGTATGATCC |
| P96 | Δ*abrB*::tet^R^ | CGATCCACATACTCCTGAGGTG |
| P97 | Δ*abrB*::tet^R^ | GAGAACAACCTGCACCATTGCAAGACATAAACATTCTCCTCCCAAGAGATAC |
| P98 | Δ*abrB*::tet^R^ | GGGATCAACTTTGGGAGAGAGTTCCAAAACCTTAAATAATCATTTCTTGTAC |
| P99 | Δ*abrB*::tet^R^ | CATTGCTCTTGTCAGTGATGC |

^1^Underlined regions in the primer sequences indicate either the location of the restriction enzyme sites used for cloning or the antibiotic cassette-joining sequence added for long-flanking homology (LFH) PCR (Butcher & Helmann, 2006).

**SUPPLEMENTAL FIGURES**

**
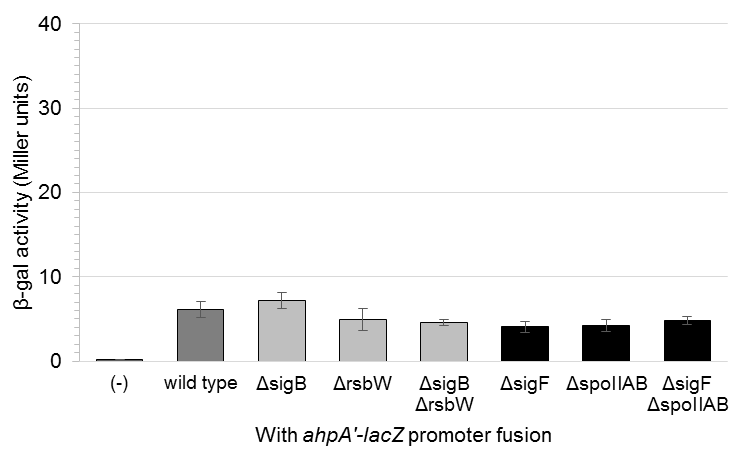
**

**Figure S1.** Expression of *ahpA* is not controlled by regulatory proteins responsible for controlling the expression of other peroxide scavenging enzymes. Shown are the expression levels of *ahpA* in strains lacking σ^B^, σ^F^, or the associated anti-σ factors during vegetative growth based on β-galactosidase activity from a transcriptional fusion of the *ahpA* promoter to *lacZ*. Strains were grown with aeration in LB broth at 37ºC to an OD_600_ of 0.8. (-) represents a wild type strain lacking any *lacZ* fusion. Error bars indicate the standard error for three independent cultures.

**
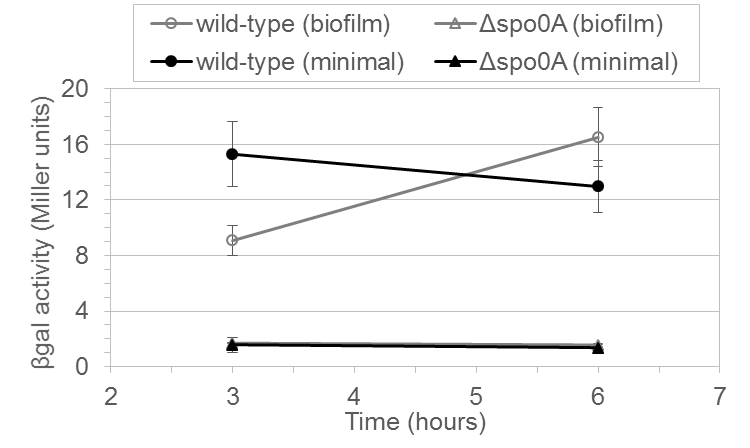
**

**Figure S2.** Expression of *ahpA* is not substantially increased upon entry into stationary phase. Shown is the expression level of *ahpA* based on β-galactosidase activity from a fusion of the *ahpA* promoter to *lacZ* (*ahpA’-lacZ*). Strains were grown for the indicated time period at 31ºC in borosilicate glass culture tubes with aeration in either biofilm promoting media (MSgg) or minimal media. Error bars indicate the standard error for three independent cultures. Cultures reached mid-log phase at 3 hours (OD_600_ = 0.4) and early stationary phase by 6 hours (OD_600_ = 1.0).

**REFERENCES**

**Bagyan, I., Casillas-Martinez, L. & Setlow, P.** **(1998).** The *katX* gene, which codes for the catalase in spores of *Bacillus subtilis*, is a forespore-specific gene controlled by sigma(F), and *katX* is essential for hydrogen peroxide resistance of the germinating spore. *J Bacteriol* **180**, 2057–2062.

**Broden, N. J., Flury, S., King, A. N., Schroeder, B. W., Coe, G. D. & Faulkner, M. J.** **(2016).** Insights into the function of a second, non-classical Ahp peroxidase, AhpA, in oxidative stress resistance in *Bacillus subtilis*. *J Bacteriol* **198**, 1044-1057.

**Butcher, B. G. & Helmann, J. D.** **(2006).** Identification of *Bacillus subtilis* sigma-dependent genes that provide intrinsic resistance to antimicrobial compounds produced by Bacilli. *Mol Microbiol* **60**, 765–782.

**Faulkner, M. J., Ma, Z., Fuangthong, M. & Helmann, J. D.** **(2012).** Derepression of the *Bacillus subtilis* PerR peroxide stress response leads to iron deficiency. *J Bacteriol* **194**, 1226–1235.

**Fawcett, P., Eichenberger, P., Losick, R. & Youngman, P.** **(2000).** The transcriptional profile of early to middle sporulation in *Bacillus subtilis*. *Proc Natl Acad Sci U S A* **97**, 8063–8.

**Fuangthong, M., Atichartpongkul, S., Mongkolsuk, S. & Helmann, J. D.** **(2001).** OhrR is a repressor of *ohrA*, a key organic hydroperoxide resistance determinant in *Bacillus subtilis*. *J Bacteriol* **183**, 4134–4141.

**Fuangthong, M., Herbig, A. F., Bsat, N. & Helmann, J. D.** **(2002).** Regulation of the *Bacillus subtilis fur* and *perR* genes by PerR: Not all members of the PerR regulon are peroxide inducible. *J Bacteriol* **184**, 3276–3286.

**Konkol, M. A., Blair, K. M. & Kearns, D. B.** **(2013).** Plasmid-encoded *comI* inhibits competence in the ancestral 3610 strain of *Bacillus* *subtilis*. *J Bacteriol* **195**, 4085–4093.

**Quisel, J. D., Burkholder, W. F. & Grossman, A. D.** **(2001).** *In vivo* effects of sporulation kinases on mutant Spo0A proteins in *Bacillus subtilis*. *J Bacteriol* **183**, 6573–6578.
